# Thermostable Xylanase Production by *Geobacillus* sp. Strain DUSELR13, and Its Application in Ethanol Production with Lignocellulosic Biomass

**DOI:** 10.3390/microorganisms6030093

**Published:** 2018-09-05

**Authors:** Mohit Bibra, Venkat Reddy Kunreddy, Rajesh K. Sani

**Affiliations:** 1 Department of Chemical and Biological Engineering, South Dakota School of Mines and Technology, Rapid City, SD 57701, USA; mohit.bibra@mines.sdsmt.edu (M.B.); r7sani@gmail.com (V.R.K.); 2 BuG ReMeDEE Consortium, South Dakota School of Mines and Technology, Rapid City, SD 57701, USA; 3 Composite and Nanocomposite Advanced Manufacturing Centre–Biomaterials (CNAM/Bio), Rapid City, SD 57701, USA; 4 Department of Chemistry and Applied Biological Sciences, South Dakota School of Mines and Technology, Rapid City, SD 57701, USA

**Keywords:** Thermostable, xylanase, *Geobacillus*, lignocellulosic biomass, ethanol

## Abstract

The aim of the current study was to optimize the production of xylanase, and its application for ethanol production using the lignocellulosic biomass. A highly thermostable crude xylanase was obtained from the *Geobacillus* sp. strain DUSELR13 isolated from the deep biosphere of Homestake gold mine, Lead, SD. *Geobacillus* sp. strain DUSELR13 produced 6 U/mL of the xylanase with the beechwood xylan. The xylanase production was improved following the optimization studies, with one factor at a time approach, from 6 U/mL to 19.8 U/mL with xylan. The statistical optimization with response surface methodology further increased the production to 31 U/mL. The characterization studies revealed that the crude xylanase complex had an optimum pH of 7.0, with a broad pH range of 5.0–9.0, and an optimum temperature of 75 °C. The ~45 kDa xylanase protein was highly thermostable with t_1/2_ of 48, 38, and 13 days at 50, 60, and 70 °C, respectively. The xylanase activity increased with the addition of Cu^+2,^ Zn^+2^, K^+,^ and Fe^+2^ at 1 mM concentration, and Ca^+2^, Zn^+2^, Mg^+2^, and Na^+^ at 10 mM concentration. The comparative analysis of the crude xylanase against its commercial counterpart Novozymes Cellic HTec and Dupont, Accellerase XY, showed that it performed better at higher temperature, hydrolyzing 65.4% of the beechwood at 75 °C. The DUSEL R13 showed the mettle to hydrolyze, and utilize the pretreated, and untreated lignocellulosic biomass: prairie cord grass (PCG), and corn stover (CS) as the substrate, and gave a maximum yield of 20.5 U/mL with the untreated PCG. When grown in co-culture with *Geobacillus thermoglucosidasius*, it produced 3.53 and 3.72 g/L ethanol, respectively with PCG, and CS. With these characteristics the xylanase under study could be an industrial success for the high temperature bioprocesses.

## Highlights:

- A thermostable xylanase production from a *Geobacillus* sp. strain DUSELR13 with t_1/2_ of 38 days at 60 °C.
- Use of the lignocellulosic biomass for the xylanase production, and ethanol production.
- Better xylan hydrolysis by DUSELR13 xylanase than the commercial counterpart at higher temperature.

## 1. Introduction

The environmental, and economic incentives associated with the utilization of lignocellulosic biomass at industrial scale drive the quest for finding new microorganisms capable of producing enzymes with better characteristics. Using substrates which create direct competition with the food resources had been discouraged at the industrial level. The abundant, economic, and ubiquitously available lignocellulosic biomass (LCB), composed of cellulose and hemicellulose can act as an ideal substrate for the industrial bioprocesses. However, the recalcitrant nature of the LCB due to structural, and chemical hardships require prior hydrolysis of cellulosic and hemicellulosic portions to obtain fermentable sugars [1,2]. Using enzymatic hydrolysis can be environmentally and economically beneficial compared to the other commonly used pretreatment methods [3].

Hemicellulose, which forms about 30–40% of the lignocellulosic biomass (LCB) is mainly constituted by xylan. Xylan is a polysaccharide made up of β-1,4-xylose units or β-1,4-mannose units, and has substitutions of arabinose, methylglucuronic acid, and acetate [4,5]. The complex chemical composition of xylan requires a concerted action of several enzymes, collectively known as hemicellulases, including endo-β-d-xylanases, β-d-xylosidases, α-l-arabinofuranosidases, α-d-glucuronidases, acetylxylan esterases, ferulic and p-coumaric acid esterases. These enzymes act synergistically on the linear chain as well as on the side chains giving xylose and xylooligosaccharides (XOS) as the end products [4,5]. Among the various enzymes required for the effective hydrolysis of xylan component of biomass, endo-1,4-β-xylanase (simply referred to as xylanase), is an essential enzyme that acts on the xylan backbone of the hemicellulose with high specificity, negligible substrate loss, and side products [4] compared to the prevalent chemical methods of hydrolysis.

The branched out application of the xylanases in paper and pulp industry, deinking, pharmaceuticals, cosmetics, food, and feed industry etc. had increased their demand, and significance in the industrial processes [4,5,6,7,8,9]. The wide operational parameters of these processes require xylanases that can perform well under the extreme conditions of temperatures, pH, salts, osmotic conditions etc. The thermostable enzymes which can operate at high temperatures ranging from 45 to 100 °C have been in demand for a long period in the industrial production, and research bioprocessing set ups. They offer several advantages such as higher mass transfer rates, and low viscosity that may lead to increased solubility of reactants and products; lower risk of contamination from mesophilic microbes, improved hydrolysis performance due to long half-lives at high temperatures, and structural and functional stability at higher temperatures [3,10].

The bioprospecting of several thermophilic bacteria belonging to genus *Geobacillus, Bacillus, Thermotoga, Thermoanerobacterium, Anoxybacillus,* and *Acidothermus* etc. had been reported that produce thermostable xylanases [5,6,8,10,11]. The thermostable xylanases obtained from the thermophilic microorganisms are more advantageous in comparison to those obtained from their mesophilic counterparts for industrial bioprocesses [3]. Among several thermophilic organisms studied for xylanase production genus *Geobacillus* had been researched extensively owing to its ability to produced highly thermostable enzymes, utilize various carbon sources [12,13]. The existence of *Geobacillus* spp. in the thermophilic areas has endowed the genus with several species the genome of which encode highly thermostable enzymes with application in several industrial bioprocesses such as the lignocellulosic biomass hydrolysis [4,5], pulp and paper production [6], bioethanol production [14] etc.

Several *Geobacillus* spp. such as *Geobacillus stearothermophilus* [15], *Geobacillus thermodenitrificans* [16], *Geobacillus* sp. strain WSUCF1 [5], and *Geobacillus thermolevorans* [6] etc. had been reported for xylanase production. Although, the *Geobacillus* spp. produce thermostable xylanases, but the duration of thermostability is very small. At 70 °C *Geobacillus thermodinitrificans* strain A333 Xyn had a t_1/2_ of 60 min [4], *Geobacillus* sp. strain TF16 had a t_1/2_ of 10 min [17], and a t_1/2_ of 8 min was observed for xylanase from *Geobacillus thermodinitrificans* strain C5 [18]. Only, the xylanase from *Geobacillus* sp. strain WSUCF1 had an exceptional t_1/2_ of 12 days at 70 °C [5]. However, the recombinant expression of the xylanase from *Geobacillus* sp. strain WSUCF1 in *E. coli* reduced the thermostability from 12 days to only 20 min at 70 °C [19]. Thus the higher thermostability was seen only when the enzyme was produced in the natural host. Several mutagenic studies had been done to increase the thermostability of the xylanases. Irfan and coworkers (2018) carried out site directed mutagenesis in the xylanase from *Geobacillus thermodinitrificans* strain C5 and increased the thermostability from 8 min to 70 min at 70 °C in a triple mutant strain [18]. The thermostability of xylanase from wild type *Geobacillus stearothermophilus* sp. strain XT6 was increased by incorporating substitutions at 13 amino acids. With amino acid substitutions the thermostability increased 52 folds from 3.5 to 182.2 min at 75 °C [20]. In addition to the mutagenic studies, literature studies also reveal several examples of improving thermostability by immobilizing the enzymes. The immobilized enzymes offers several advantages such as improved selectivity, structural stability, reduced sensitivity, and reduced costs due to easy enzyme recovery etc. when compared to the soluble enzymes [17]. With additional benefits of enzyme thermostability the process of immobilization becomes more lucrative. In a study carried out to increase the thermostability of the xylanase enzyme from *Geobacillus* sp. strain TF16 by immobilizing the enzyme with chitosan resulted in enzyme retaining 45% of its activity after 6 h at 55 °C compared to 2 h with the free enzyme [17]. Thus, thermostability of the hydrolytic enzymes is one highly sought characteristic as is evident from the recent works focused on increasing the thermostability of the enzymes [21,22,23]. However, the anthropogenic measures of increasing thermostability till date are no match for the natural thermostability as observed in the crude xylanase obtained from the wild-type *Geobacillus* sp. strain WSUCF1. Hence, bioprospecting wild type strains that can produce highly thermostable enzymes is very important.

*Geobacillus* sp. strain DUSELR13 is one such *Geobacillus* sp. which earlier had been reported to produce cellulase with thermostable characteristics [24]. We report here the production of a highly thermostable endoxylanase from *Geobacillus* sp. strain DUSELR13. The production of the xylanase from DUSELR13 was optimized taking into account several physical and biochemical factors. To increase the production further, statistical optimization was carried. A central composite design (CCD) was created in response surface methodology to increase the xylanase production. A comparative analysis of the DUSELR13 xylanase to a commercial enzymatic formulation was further researched to find its suitability on the industrial scale. The xylanase produced was characterized to find the optimum pH, temperature, thermostability, and effect of the metal ions. The xylanase production was further carried with the lignocellulosic biomass to improve the economics of the enzyme production. Finally, a co-culture study was done to study the application of the enzymatic hydrolysis of LCB by *Geobacillus* sp. strain DUSELR13 to produce ethanol with *Geobacillus thermoglucosidasius*.

## 2. Materials and Methods

### 2.1. Microorganism and Enzyme Assay

The *Geobacillus* sp. strain DUSELR13, previously known as the *Bacillus* sp. strain DUSELR13., was isolated from the deep biosphere of Homestake gold mine, Lead, South Dakota (44°21′3″ N, and 103°45′57″ W) [24]. It was grown in 90 mL of the minimal media developed previously in our lab [25] with 0.5% (*w*/*v*) of xylan as the carbon and energy source in 500-mL Erlenmeyer flasks. The composition of the minimal media per liter is: 0.1 g nitrilotriacetic acid, 0.05 g CaCl_2_·2H_2_O, 0.1 g MgSO_4_·7H_2_O, 0.01 g NaCl, 0.01 g KCl, 0.3 g NH_4_Cl, 0.005 g methionine, 0.2 g yeast extract, 0.01 g casamino acids, 1.8 g of 85% H_3_PO_4_, 1 mL FeCl_3_ solution (0.03%), and 1 mL of Nitsch’s trace solution. The pH of the medium was kept at 7.0 using 6 M NaOH. The flasks were incubated in a shaker incubator at 60 °C, and 150 rpm for 96 h. A control flask designated as the organism free control was also kept under the similar conditions. After 12 h, 3 mL samples were removed aseptically, and analyzed for growth by measuring OD_600nm_. The collected samples were centrifuged at 4 °C and 10,000× *g* for 10 min, and the supernatant was retained for the endoxylanase activity described below.

### 2.2. Enzyme Assay

For endoxylanase enzyme assay, performed as per Bailey et al. [26], the reaction mixtures contained 1.8 mL of 1% (*w*/*v*) birchwood xylan (Sigma-Aldrich, St. Louis, MO, USA) in phosphate buffer (100 mM, pH 7.0) and 0.2 mL of an appropriate dilution of the supernatant prepared for HPLC analysis containing enzyme. The enzyme-substrate reaction was carried out at 60 °C for 10 min. The reaction was stopped by the addition of 3.0 mL 3,5-Dinitrosalicylic acid (DNSA) solution, boiled for 10 min, and then cooled on ice for color stabilization. The optical absorbance was measured at 540 nm, and the amounts of liberated reducing sugar (xylose equivalents) was estimated against the standard curves for xylose. One unit of xylanase enzyme was defined as the amount of enzyme that releases 1 µmol of xylose per minute under reaction conditions. 

### 2.3. Cell Morphology

The cell morphology was determined using the Scanning electron microscopic (SEM). For sample preparation, 5 mL of culture solution was taken in 15 mL centrifuge, and centrifuged at 10,000 rpm for 10 min. The pellet was washed with PBS (pH 7.2, 100 mM) three times. A mixture of glutaraldehyde and cacodylate buffer 100 mM pH 7.2 was added in a ratio 1:9 (*v*/*v*), and mixed gently to fix the samples. Afterwards, the samples were kept on the ice for 45–60 min. The suspension was centrifuged at 6000 rpm for 5–10 min. After centrifugation the supernatant was removed and the pellet was dehydrated using ethyl alcohol. The concentration of the ethyl alcohol was increased in a graduated manner: 30%, 50%, 70%, 90% and 100%. The samples were centrifuged at each step involving addition of ethyl alcohol. With each centrifugation step the supernatant was removed, and after final centrifugation with 100% alcohol the pellet was mounted on an aluminum stub and allowed to dry before microscopic analysis.

### 2.4. Optimization Studies for Xylanase Production

#### 2.4.1. One Factor at a Time

To find the parameters affecting the xylanase production, different pH’s (5.0–9.0 with an increment of 1.0), temperatures (50–70 °C with an increment of 5 °C), xylan concentrations (0.1, 1.0, 2.0, 3.0, and 4.0% (*w*/*v*)), and nitrogen sources (yeast extract, urea, tryptone, beef extract, and peptone 0.05% (*w*/*v*)) were chosen. The studies were done using one factor at a time (OFAT) approach. In OFAT approach, one factor was optimized at a time, and that value was used to find the optimized value for the other factor. The pH value was selected first and was used to find out the optimal temperature, followed by the xylan concentration and selection of the nitrogen source, for the crude xylanase production. The reaction set for the enzyme optimization studies was prepared as described above.

#### 2.4.2. Response Surface Methodology

Response Surface Methodology (RSM) was used to optimize the fermentation parameters to enhance, and find out the effect of two factor interactions on the extracellular xylanase production, using Design Expert Version 11.0.0 (Stat-Ease Inc., Minneapolis, Minnesota, MN, USA) statistical software. Four variables: temperature, pH, xylan, and tryptone, were chosen for the statistical optimization with all the variables set at a central coded value of zero. Each variable was studied at five different levels (−α, −1, 0, +1, +α) as shown in Table 1. The design included six center points with an alpha value of ±0.5. All the factorial points, and axial points were studied in triplicates giving a total number of runs 78, as per the combination expression m × 2^k^ + m × 2 × k + n where ‘m’ is the number of replicates, ‘k’ is the number of variables, and ‘n’ is the number of center points. Quantitative data generated from these experiments shown in Table 2 was subjected to analysis of regression through RSM to solve multivariate equations. The effects of variables to the response were analyzed by using a second-order polynomial equation:

(1) $$\begin{array}{c} Y=\beta_{0}+\beta_{1}A+\beta_{2}B+\beta_{3}C+\beta_{4}D+\beta_{11}A^{2}+\beta_{22}B^{2}+\beta_{33}C^{2}+\\ \beta_{44}D^{2}+\beta_{12}\mathrm{AB}+\beta_{13}\mathrm{AC}+\beta_{14}\mathrm{AD}+\beta_{23}\mathrm{BC}+\beta_{24}\mathrm{BD}+\beta_{34}\mathrm{CD} \end{array}$$

where, Y is the predicted response, A, B, C, and D, are the coded levels of the independent parameter, β_0_ represents the intercept, β_1_, β_2_, β_3_ and β_4_ are linear effect coefficients; β_11_, β_22_, β_33_ and β_44_ are the quadratic effect coefficients, β_12_, β_13_, β_14_, β_23_, β_24_, and β_34_ are the interaction effect coefficients. The statistical significance of the model was estimated by analysis of variance (ANOVA) with *p*-value < 0.05 i.e., above 95% confidence level and insignificance of lack of fit test.

The quality of the model developed was evaluated by R-squared values i.e., coefficient of determination: adjusted R^2^ and predicted R^2^. The fitted polynomial equation was expressed as 3D surface plots to illustrate the relationship between the responses and any two variables to be optimized, keeping the other variables at central positions. Further, numerical optimization method was used for obtaining the optimal solution by keeping the desirability at maximum. The model obtained was validated by running the experiment based on the optimum values obtained for the variables.

### 2.5. Xylanase Characterization

Sodium dodecyl sulfate-polyacrylamide gel electrophoresis (SDS-PAGE) was performed to find the molecular weight of the protein, as described by Laemmli [27]. Ten milliliters of the supernatant from Erlenmeyer flasks with xylan and PCG was concentrated (5-times) using Amicon Ultra-15-Millipore (10 kDa cut off). Ten microliters of the concentrated protein was mixed with ten microliters sample buffer (2X). The composition of the sample buffer (4X) per liter was: 250 mL^−1^ M Tris-HCl (pH 6.8), SDS-100 g, 0.1% bromophenol blue (*w*/*v*)-80 mL, glycerol-400 mL, 14.3 M β-mercaptoethanol-200 mL, and distilled water to make the volume 1 L. The enzyme extracts containing equal amounts of protein (50 micrograms) were resolved on 10% SDS-PAGE at constant voltage (150 V) till the dye front reached the bottom of the gel. For zymogram, the gel was renatured by washing successively for 30 min with: 20% isopropanol in phosphate buffer saline (PBS, 100 mM, pH 5.9), 8 M urea in PBS, and PBS (pH 5.9) three times. The re-natured gel was placed in sodium phosphate buffer (50 mM, pH 7.0) for 15 min and subsequently, Beechwood xylan (prepared in 50 mM sodium phosphate buffer, pH 7.0), was overlaid on the gel. The gel was incubated at 60 °C for 30 min. This was followed by staining with Congo red (1 mg/mL) for 30 min, and de-staining with 1 M NaCl in PBS until clear bands indicating xylanase activity were visible. The SDS-PAGE gels loaded with the PCG samples were treated with the Silver Stain Plus kit (BioRad, Hercules, CA, USA) as per the manufacturer’s instruction.

One percent (*w*/*v*) Beechwood xylan was used to determine the relative xylanase activity at various pHs. The pH optimum (pH_opt_) of crude xylanase was estimated by testing enzyme activity in the pH range of 3.0–10.0 using different assay buffers, citrate buffer (100 mM, pH 3–6), phosphate buffer (100 mM, pH 6–7.5), Tris-HCl (100 mM, pH 7.5–9), and glycine-NaOH buffer (100 mM, pH 8.6–10) at 60 °C for 10 min. The enzyme activity obtained at the pH_opt_ was used to calculate the relative enzyme activity at other pHs. The optimum pH of 7.0 was used to determine the optimum temperature for the crude xylanase.

The temperature optimum (T_opt_) for crude xylanase was obtained by performing the enzyme assays at different temperatures. The experiments were carried out at temperatures: 25 °C, 37 °C, and a range of 50–100 °C with an increment of 5 °C under assay conditions as described above. The thermostability of xylanases was assessed by incubating the enzyme at different temperatures 50–100 °C with increments of 10 °C for a period of 80 days. The effect of metal ions on the enzyme activity was determined for Cu, Co, Ca, Mg, Zn, Mn, Na, K, and Fe at 1 mM and 10 mM concentrations. The enzyme solution in 100 mM phosphate buffer (pH—7.0) was doped with the metal salt, and incubated at 60 °C for 1 h. The sampling was done at predetermined time intervals over the period of incubation. The residual activities were determined under optimum pH and temperature conditions using the DNSA method as described above. Throughout the optimization studies, the optimum activity was assumed to be 100%, and the relative enzyme activities were calculated against it. While for the metal ion characterization study, the test study where no metal ion was added was considered as the 100% activity, and all the activities were calculated against it.

### 2.6. Hydrolysis of Birchwood Xylan

The hydrolysis of Birchwood xylan was carried out in 100 mL conical flask containing 50 mL sodium phosphate buffer (50 mM, pH 7.0) (buffering agent), 1 g xylan, 0.03% (*w*/*v*) sodium azide (preservative), sucrose 150 mM (stabilizer) and 20 U crude xylanase/g xylan. The hydrolysis was performed for 48 h at different temperatures (50–75 °C with an increment of 5 °C) with a rotating speed of 150 rpm. Hydrolysis of xylan was also compared using Cellic HTec2 (Novozymes, Franklin, NC, U.S.A.), and Accelerase XY (DuPont, Palo Alto, CA, U.S.A.) with similar enzymatic units as of DUSELR13. A pH of 5.0, and different temperatures in the stable operational range (50–75 °C) of commercial counterparts, was used to compare the hydrolytic potential with the xylanase from *Geobacillus* sp. strain DUSELR13 xylanase. The amount of reducing sugar was measured by HPLC (Shimadzu LC20; Columbia, MD, USA) equipped with a 300 × 7.8 mm Aminex HPX-87H column (Bio-Rad, Torrance, CA, USA). One mL of the samples was removed from the Erlenmeyer flasks, and centrifuged at 10,000 rpm for 10 min. The supernatant obtained was removed and filtered using 0.2 μm pore size membrane filters (Gelman Acrodisc, Sigma Aldrich, St. Louis, MO, USA). The filtered samples were automatically injected onto a heated column (50 °C) and eluted at 0.45 mL/min using 5 mM H_2_SO_4_ as the mobile phase in HPLC.

### 2.7. Enzyme Production with Lignocellulosic Biomass

For enzyme production with lignocellulosic biomass (LCB) 1% (*w*/*v*) of untreated and mechanically pretreated (Prairie cordgrass (PCG) and corn stover (CS)) were used as the substrate. One hundred ml of the minimal media, supplemented with 10 g/L tryptone, and the pH set to 6.5 using 6 M NaOH, was added to the 500 mL Erlenmeyer flasks. The flasks were autoclaved at 121 °C and 15 psi for 15 min. After autoclaving 1% (*w*/*v*) of insoluble LCB obtained as described by Bibra et al. (2018) was added to the flasks [28]. The flasks were kept at 59 °C for 96 h. After 12 h 3 mL samples were removed aseptically. The collected samples were centrifuged at 4 °C and 10,000× *g* for 10 min, and the supernatant was used for measuring the endoxylanase activity (explained above). 

### 2.8. Co-Culture of Geobacillus sp. strain DUSELR13 and Geobacillus thermoglucosidasius for Ethanol Production

The lab strain *Geobacillus* sp. strain DUSELR13 capable of producing thermostable ligninolytic enzymes was used for the ethanol production with ethanol producing strain *Geobacillus thermoglucosidasius* (ATCC 43742). Five percent (*w*/*v*) of insoluble mechanically treated lignocellulosic biomass (prairie cord grass-PCG, and corn stover-CS) were added to the 100 mL of the minimal media in 500 mL Erlenmeyer flasks. The pH of the media was adjusted to 6.5 using 6 M NaOH, and followed by the addition of 10% (*v*/*v*) of the actively growing culture of *Geobacillus* sp. strain DUSELR13 to the Erlenmeyer flask. The flasks were kept at 59 °C and 150 rpm for 36 h for enzyme production. After 36 h 20 mL of fresh media (5X) was added to the Erlenmeyer flask for ethanol production, and pH was adjusted to 7.0 using 6 M NaOH. Ten percent (*v*/*v*) of the actively growing culture of *Geobacillus thermoglucosidasius* was added to the Erlenmeyer flask, and the flasks were capped by polyvinyl stoppers. The amount of ethanol and volatile fatty acids produced were measured with HPLC as explained before [25]. The ethanol yield ‘Y_P/S_’ (g/g) and ethanol productivity ‘q_p_’ (g/L/h) were measured using the equation below:

(2) $$Y_{P/S}\;\left(g/g\right)=\frac{\mathrm{Amount}\;\mathrm{of}\;\mathrm{ethanol}\;\left(g\right)}{\mathrm{Amount}\;\mathrm{of}\;\mathrm{susbtrate}\;\mathrm{utilized}\;\left(g\right)}$$

(3) $$q_{P}\;\left(g/L/h\right)=\frac{\mathrm{Amount}\;\mathrm{of}\;\mathrm{ethanol}\;\mathrm{produced}\;\left(g\right)}{\mathrm{Volume}\;\left(L\right)*\mathrm{Time}\;\left(\mathrm{hours}\right)}$$

### 2.9. Material and Energy Balance

The material balance was carried out in terms of mass balance for PCG, and CS. The energy efficiency for conversion of PCG to ethanol was calculated using the following equation:

(4) $$\eta =\frac{\mathrm{xEthanol}*\mathrm{EEthanol}*100}{\Delta H_{C6,C5\mathrm{LCB}}*M_{\mathrm{LCB}}}$$

where η represent energy efficiency, xEthanol represents the amount of ethanol produced in moles, EEthanol represents the energy density of ethanol (28.6 MJ/kg), ΔH represents the heat of combustion for hexose and xylose portion of the lignocellulosic biomass, and M_LCB_ represents the amount of lignocellulosic biomass used.

## 3. Results and Discussion

### 3.1. Microorganism

**Figure 1:**
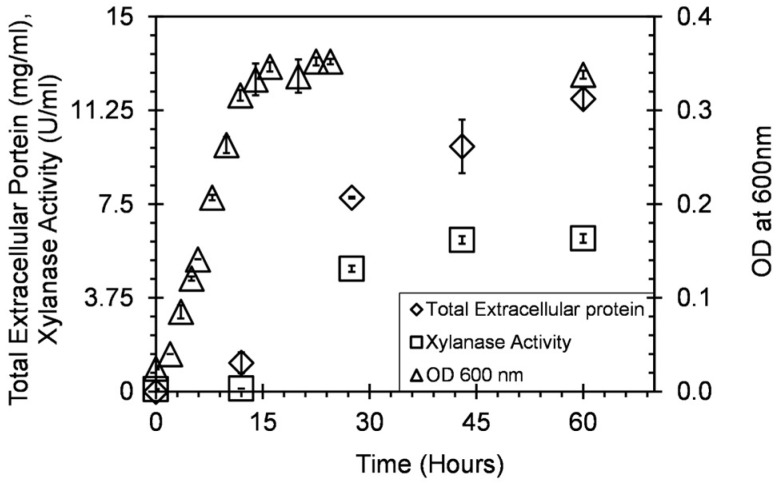
The growth, total extracellular protein, and xylanase activity profile of *Geobacillus* sp. strain DUSELR13 grown on 0.05% (*w*/*v*) xylan as the substrate at 60 °C. The values are the means of three set of experiments and the error bars represent ± SD of the means with *n* = 3.

The preliminary experiments showed that *Geobacillus* sp. strain DUSELR13 was capable of producing xylanase. The Figure 1 shows the endoxylanase activity, total extracellular protein, and OD_600nm_ with time. *Geobacillus* sp. strain DUSELR13 showed a typical growth curve achieving maximum OD_600nm_ ~0.355 absorption units (A.U.) in 15 h during the exponential phase (Figure 1). After that the OD_600nm_ became constant, signifying a stationary phase. When grown on 0.05% (*w*/*v*) Beechwood xylan, DUSELR13 produced 6 U/mL of the thermostable endoxylanase (Xyl) activity and a total extracellular protein of 12.7 mg/mL (Figure 1). The extracellular protein and endoxylanase activity on the other hand still increased after 14 h, thus ruling out any correlation between the growth and the extracellular protein production and the endoxylanase activity. Purohit and coworkers (2017) also observed that the maximum enzyme activity was observed after 24 h when the maximum growth for *Acinetobacter putii* MASK25 was achieved [29]. It was also reported by Shulami and coworkers (2014) that *Geobacillus stearothermophilus* produced xylanase during the late exponential phase, where the cells could survive the nutrient limiting conditions, and produced more xylanase to hydrolyze the xylan and generate more sugar [15]. 

**Figure 2:**
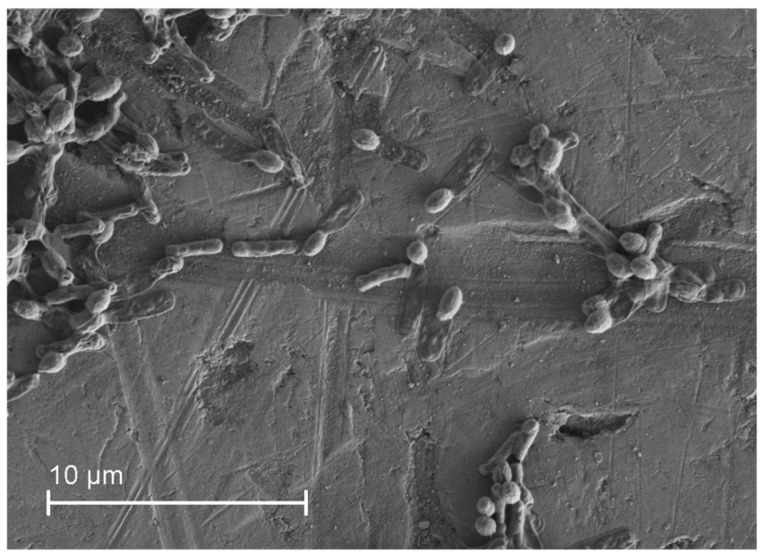
SEM image of *Geobacillus* sp. strain DUSELR13 during the late stationary phase. The majority of the cells show appear as flaccid with the presence of the terminal endospores.

After 42 h till end, no change in the endoxylanase activity was observed, whereas the extracellular protein still increased which could be due to release of the cell protein after cell lysis during this phase. As the organisms approach towards the end of the stationary phase with nutrient limitation becoming more prominent, they tend to adopt protectionary mechanisms such as the endospores formation. With endospore formation the cell metabolism stops. In the current study, the scanning electron microscopy (SEM) image of the culture during the stationary phase showed rod shaped organism with endospores at the terminal position (Figure 2). This observation regarding formation of endospores during late stationary phase further substantiates the reason for no change in the xylanase activity in the later stages of the bacterial growth.

### 3.2. Optimization of Endoxylanase Production

The secretome obtained during the growth cycle of an organism consists of several proteins, and xylanase might represent a smaller fraction of that secretome. Hence, to increase the production of the endoxylanase several factors were chosen and studied to find the optimal values for xylanase production. 

#### 3.2.1. One Factor at a Time

##### pH

**Figure 3:**
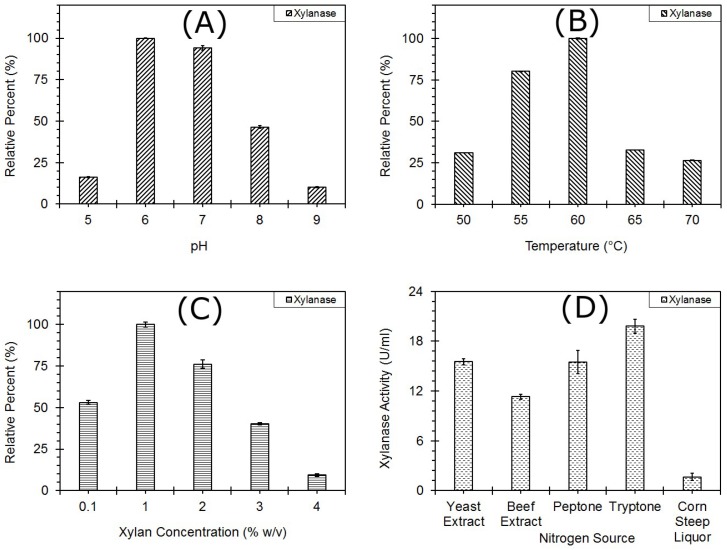
(**A**) The effect of growth pH profile on the xylanase activity of *Geobacillus* sp. strain DUSELR13. The maximum enzyme activity (8.54 U/mL) is taken as 100%; (**B**) the effect of growth temperature profile on the xylanase activity of *Geobacillus* sp. strain DUSELR13. The maximum enzyme activity (9.23 U/mL) is taken as 100%; (**C**) the effect of xylan concentration on the xylanase activity of *Geobacillus* sp. strain DUSELR13. The maximum enzyme activity (17.4 U/mL) is taken as 100%; and (**D**) the effect of nitrogen source on the xylanase activity of *Geobacillus* sp. strain DUSELR13. Values shown were the mean of triplicate experiments and the error bars represent ± SD of the means with *n* = 3.

The strain DUSELR13 was grown at different pHs (5–9, with an increment of 1.0). The pH 6.0 (8.54 U/mL-100%) was found to be optimal for the enzyme production. Figure 3A shows the relative percent of the xylanase activity at different pHs. The order of pH for the relative xylanase activity at different pH for the production was 6(100%) > 7(94.14%) > 8(46.3%) > 5(16.05%) > 5(10.12%). The xylanase production decreased as the growth pH deviated from the pH_opt_. The relative activity of the xylanase decreased by 40–50% at pH’s 5, 8, and 9. The pH 6.0 was also found to be optimum for xylanase production by *Geobacillus stearothermophillus* strain KIGBE-IB29 [30] and *Bacillus altitudinis* strain DHN8 [31]. On the contrary, *Geobacillus* sp. strain WSUCF1 exhibited a pH_opt_ of 6.5 for the xylanase production [5]. The external pH affects the transport of the enzymes and chemical products across the membrane [5]. Hence finding a pH value where the extracellular secretion of protein is favored is important.

##### Temperature

When grown at different temperatures (50–70 °C, with an increment of 5 °C), 60 °C was found to be the T_opt_ for xylanase production (9.23 U/mL-100%) for the strain DUSELR13. The enzyme activity increased from 50 to 60 °C, but decreased from 60 °C to 70 °C, The relative xylanase activity was measured against activity obtained at T = 60 °C, and the order of xylanase activity was 60(100%) > 55(80.13%) > 65(32.7%) > 50(31.12%) > 70(26.44%) °C (Figure 3B). The temperature 60 °C has also been reported as T_opt_ for the endoxylanase production in *Geobacillus* sp. strain WSUCF1 [5] and *Geobacillus steareothermophillus* [15]. Different T_opt_’s have also been reported for endoxylanase production by several other thermophilic species: *Anoxybacillus flavithermus* Strain TWXYL3 had a T_opt_ of 65 °C [32], whereas *Bacillus amyloliquefaciens* showed T_opt_ of 50 °C for the xylanase production [23]. The production of the enzymes at a high temperature offers advantages such as no contamination and reduced viscosity [3]. A temperature higher than the T_opt_ is more detrimental than a temperature lower than the T_opt_. High temperatures such as 70 °C can cause the denaturation of several key enzymes required for the cell survival, resulting in reduced growth and lower enzymatic activity. Hence, low xylanase activity was observed at 70 °C. As reported previously, the extracellular protein secretion involves several membrane associated proteins, and is affected by the rate of the folding of such proteins, and the dynamics of complex formation/dissolution within the cell envelope dependent [33]. As the temperature deviates from the T_opt_ production it directly affects all these processes, affecting the extracellular protein secretion, and the enzyme activity. 

##### Xylan Concentration

An optimum substrate concentration ensures to provide the amount of carbon and energy required for biochemical and physiological events. Xylan concentration 0.1, and 1–4% (*w*/*v*) were used to determine the best possible xylan concentration for xylanase production. It was found that a xylan concentration of 1% (*w*/*v*) gave maximum xylanase activity (17.4 U/mL). The xylan concentration followed a trend of 1(100%) > 2(76.1%) > 0.1(53%) > 3(40.1%) > 4(9.4%). With increase in the xylan concentration from 1 to 4%, the xylanase activity decreased. A higher concentration of the substrate can cause substrate inhibition, and osmotic effects, resulting in reduced physiological and biochemical activity [25]. On the contrary, a lower concentration of the substrate is unable to provide the required amount of carbon and energy sources, and hence resulted in lower xylanase activity. Kumar and Satyanarayana (2014) also showed reduction in the xylanase activity with increase in the substrate concentration [34]. As the amount of wheat brain increased from 10 g to 40 g, the xylanase activity decreased from 4768 to 3878 U/g dbb [34]. Similar results were also observed by Bibi and coworkers (2014) where with increase in the xylan concentration from 0.5% to 3% (*w*/*v*) 50% of the xylanase activity was reduced [30].

##### Nitrogen Source

An optimal source of nitrogen is required for the synthesis of biomolecules e.g., proteins and DNA in a growing cell. Among the different organic nitrogen sources tested, tryptone was found to be having maximum effect on the xylanase production (Figure 3C). The order of xylanase production was tryptone (19.8 U/mL) > yeast Extract (15.5 U/mL) > peptone (15.5 U/mL) > beef extract (11.3 U/mL) > corn steep liquor (1.64 U/mL). The organic nitrogen sources are a good source of amino acids and vitamins and sources such as tryptone and yeast extract have been very commonly used in the fermentation process. Tryptone was also found to be the best organic nitrogen source for xylanase production with *Bacillus subtilis* sp. BS04 [11] and *Geobacillus thermolevarans* [35], however, yeast extract was preferred nitrogen source for xylanase production by *Actinomadura geliboluensis* [36], and beef extract for *Bacillus pumilis* SV-85S [7]. The corn steep liquor, a byproduct from wet corn milling, contains residual sugars from refining, and can be a good source of nitrogen, amino acids, vitamins, and minerals. However, in the present study it showed lower xylanase production in comparison to other nitrogen sources (Figure 3D), the reason for which is unknown. The nitrogen sources which support the bacterial growth better, aid in higher enzyme production. Thus, the presence of an optimal nitrogen source is an essential requirement for the better growth, and hence better enzyme production.

#### 3.2.2. Response Surface Methodology

**Figure 4:**
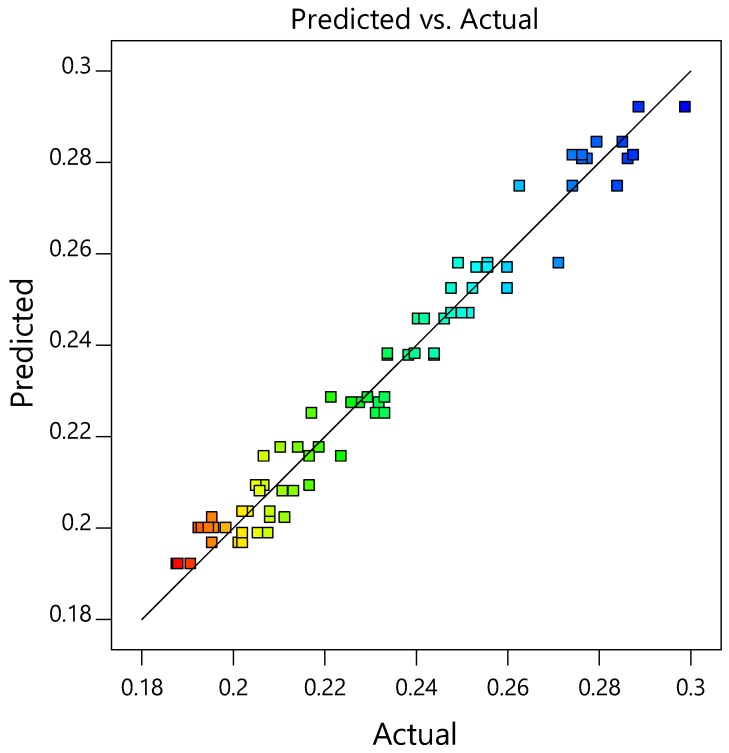
Surface response plot for predicted vs. actual values. The concentration of the data points near the straight line shows high correlation, and adequate precision. The blue color represents the minimum value, whereas the red color represents the maximum value. The rest of the color represents a range between the colors red, and blue. Each square represents the experimental value obtained from the runs.

A quadratic model based on central composite design (CCD) was developed in response surface methodology (RSM) to find the optimum parameter value for the variables mentioned in Table 1. The CCD matrix of the independent variable in coded form is shown in Table 2 with the experimental and predicted values. The analysis of variance of the quadratic model showed that the model was significant based on the *p* values, and F test (Table 3). The F value for the model was 146.69, with *p* value < 0.0001 showing high significance of the model. The model values A, B, C, D, AB, AD, A^2^, B^2^, C^2^, and D^2^ were considered significant as the *p* values for these terms in the model was <0.05. A transformation of inverse square root was applied as suggested by the simulation to fit the data in the quadratic design model. The correlation coefficient value was close to 1, indicating high correlation between the experimental and predicted values (Figure 4). A difference of less than 0.2 was obtained between the Adjusted R^2^: 0.9636 and Predicted R^2^: 0.9538 with an adequate precision of 38.12. A low coefficient of variance value (C.V. %)-2.56 indicated adequate precision and applicability of the model to navigate the design space. The Lack of Fit value −1.61, indicated that the lack of fit was not significant relative to the pure error, and the developed model is fit for predicting the enzyme activity using these variables. The model equation obtained for xylanase activity prediction in coded terms is as follows:

(5) $$\begin{array}{c} \frac{1}{\surd Y}=0.2001+0.0189\;A+0.0070\;B+0.004\;C-0.0068\;D-\\ 0.0024\;\mathrm{AB}+0.0006\;\mathrm{AC}+0.0017\;\mathrm{BC}+0.0025\;\mathrm{BD}+0.0016\;\mathrm{CD}+\\ 0.0248A^{2}+0.0231B^{2}+0.0232C^{2}-0.0181{\;D}^{2} \end{array}$$

where A, B, C, D, and Y represent temperature, pH, xylan, tryptone, and xylanase activity respectively.

**Figure 5:**
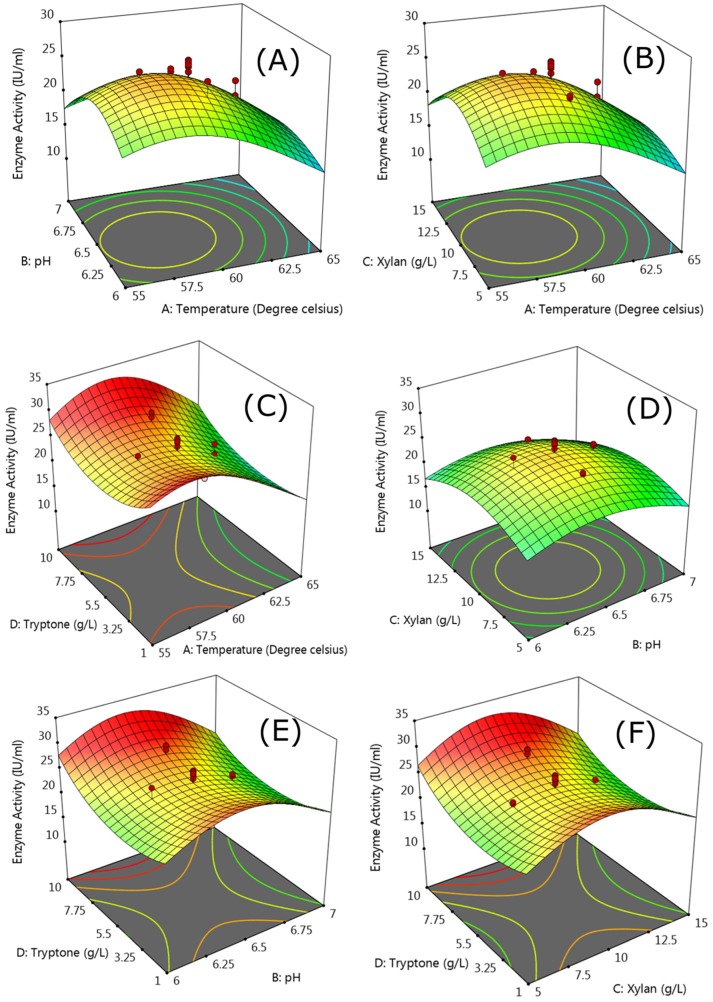
Response surface 3D response plots showing interaction between (**A**) temperature and pH, (**B**) temperature and xylan, (**C**) temperature and tryptone, (**D**) pH and xylan, (**E**) pH and tryptone, and (**F**) xylan and tryptone. The green color represents the minimum value whereas the red color represents the maximum values. The points above or below the response surface area respectively represent the values which are higher or lower than the predicted response value for the enzymatic activity.

The Figure 5A shows that a pH from the range 6.25 to 6.5 exhibited higher xylanase activity when the temperature was in the range of 57.5 to 60. Similarly Figure 5B showed that a xylan concentration of 10g/L gave higher enzymatic activity near the optimum temperature of 60 °C. As the temperature increased >60 °C the xylanase activity decreased with other variables. However, interesting results were obtained with tryptone which showed the increase in enzymatic activity at higher and lower concentration i.e., 1g/L, and 10 g/L, while interacting with the other variables (Figure 5C,E,F). A point prediction with numerical optimization showed a xylanase activity of 32.45 U/mL with 8.47 g/L xylan, 10 g/L tryptone, and pH 6.48 at a temperature of 58.9 °C. The post analysis run for the model verification gave an enzymatic activity of 31 U/mL, falling within >95% of the predicted value by the quadratic model depicting the usefulness, and precision of the model. Statistical optimization is a very powerful tool in determining the process parameter values for increasing the desired product yield. Khusro and Coworkers (2016) also obtain 3.7-fold increase in the xylanase production after statistical optimization [37]. In a similar fashion Kumar et al. (2017) increased the xylanase production from 61.09 U/mL to 119.91 U/mL via statistical optimization [38]. They also optimized the selected variables with OFAT approach, before using the statistical optimization later. A combination method of OFAT, factorial design, and/or placket burman designs can help in obtaining the preliminary data which can be used for optimization later. In the present study with statistical optimization, 5.2 times increase in the xylanase activity was observed. 

### 3.3. Enzyme Characterization

**Figure 6:**
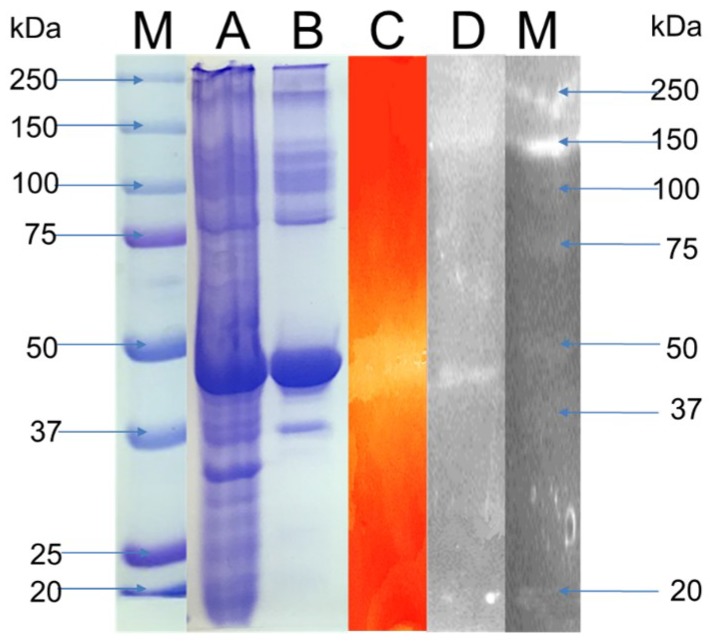
SDS PAGE, and zymogram analysis of the xylanase resolved on the 12.5% polyacrylamide gel. Lane M: Biorad precision plus standard protein marker; lane A: Crude enzyme; lane B Concentrated supernatant; lane C: Zymogram for the xylanase; lane D: SDS PAGE analysis of the xylanase grown on PCG with silver staining.

The characterization studies were carried to find the biochemical properties of the crude enzyme. For SDS-PAGE and zymogram analysis supernatant obtained after the centrifugation of sample was used. The supernatant was concentrated using the 10 kDa centrifuge filter. The SDS-PAGE and zymogram analysis is shown in the Figure 6. The PAGE analysis of total cell proteins showed several bands (Lane A), in comparison to the supernatant collected after the centrifugation where fewer bands were observed (Lane B). A thick prominent band was observed between the molecular weight 37 and 50 kDa in both the lanes A and B. The band position suggested that the molecular weight of the protein was closer to 45 kDa. In addition, a clear single smear against a red background falling in the similar weight category was observed during the zymogram analysis, when the PAGE gels were overlaid with xylan (Lane C). The two analyses confirmed that the band corresponded to the protein xylanase. The past literature studies had shown that several xylanases from the *Geobacillus* spp. and other microorganisms had molecular weight similar to the xylanase under study. The xylanase from *Geobacillus thermodenitrificans* A333 had a molecular weight of 44 kDa [4], and the molecular weight of the xylanase from *Geobacillus thermolevorans* was ~45 kDa [6]. A thermostable xylanase from fungus *Malbranchea pulchella* had a molecular weight of 41.6 kDa [8], while another thermostable xylanase from *Humicola insolens* Y1 [39], had a molecular weight of 44 kDa. However, the molecular weight of a highly thermostable xylanase obtained from the *Geobacillus* sp. strain WSUCF1 was 37 kDa [19]. The SDS-PAGE analysis of the crude xylanase obtained from the growth of DUSEL R13 on PCG showed the presence of several bands of molecular weight ~160, 45, and 20 kDa after silver staining (lane D). The silver staining of the PAGE gels allow to identify even very small concentrations of the proteins because of its greater sensitivity [40]. The presence of bands other than 45 kDa band showed that additional proteins were expressed when DUSELR13 was grown on PCG, which were absent when grown on the xylan. Thus a concerted action of several proteins aid in the growth of the DUSELR13 on the lignocellulosic biomass.

**Figure 7:**
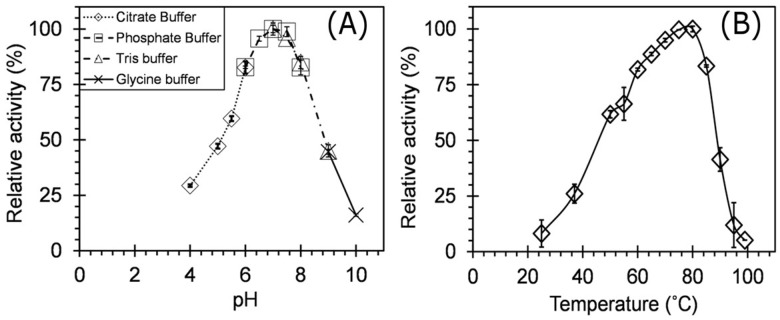
The effect of pH (**A**), and temperature (**B**) on the xylanase activity of *Geobacillus* sp. strain DUSELR13. The maximum enzyme activity is taken as 100%. The values shown are the mean of triplicate experiments and the error bars represent ± SD of the means with *n* = 3.

Further characterization of the crude xylanase revealed that the maximum xylanase activity was observed in the 100 mM phosphate buffer at pH 7.0, with activity in broad pH range from 5.0–9.0. The xylanase from DUSELR13 retained >45% activity at a pH range of 4.0–9.0 and >82% activity in a pH range of 6.0–8.0 9 (Figure 7A). Several other *Geobacillus* spp. had a variable pH_opt_ and a wide pH range for the thermostable xylanases. *Geobacillus* sp. strain WSUCF1 had a pH_opt_ of 6.5 and range: 4.5–8.5 [5]; *Geobacillus* sp. strain TC-W7: pH_opt_: 8.2 and range: 5.2–10.2 [41]; *Geobacillus thermonitirificans* strain A333: pH_opt_: 7.5, and range: 7.5–10.0 [4]; and *Geobacillus thermodenitrificans* strain T12: pH_opt_: 6.0, and range: 3.0–9.0 [16] respectively. The broad pH range applicability of the xylanase under current study can be very advantageous for biofuel, paper, and pulp, xylitol, and many other important industries which require direct use of xylanase under different pH conditions [3,42]. Table 4 shows the comparison of characteristics of several thermostable xylanases.

To find the optimum temperature for the xylanase from DUSELR13, the activity was assayed at different temperatures (25, 37, 50–100 °C with an increment of 5 °C). The characterization study for the temperature revealed that 75 °C was the T_opt_ for the xylanase from DUSELR13 (Figure 7B). The xylanase retained more than 60% of the enzyme activity at temperatures between 50 and 85 °C. Around 50% of the tested points fell between temperature ranges of 60–85 °C and accounted for more than 75% of the relative xylanase activity. The lowest relative xylanase activity was observed at 100 °C followed by 25 > 95 > 37 °C suggesting that 25 and 37 °C were too low for the enzyme activity, while 95 and 100 °C were too high temperatures for the enzyme activity. Several thermostable xylanases have T_opt_ similar or closer to strain DUSELR13 e.g., *Geobacillus* sp. strain TC-W7 had a T_opt_ of 75 °C [41]; *Geobacillus thermonitirificans* strain A333, *Geobacillus* sp. strain WSUCF 1 showed a T_opt_ of 70 °C [4], and *Malbranchea pulchela* had a T_opt_ of 80 °C [8]. The results support the ability of the DUSELR13 xyalanse to perform in the industrial process which operate at high temperatures.

**Figure 8:**
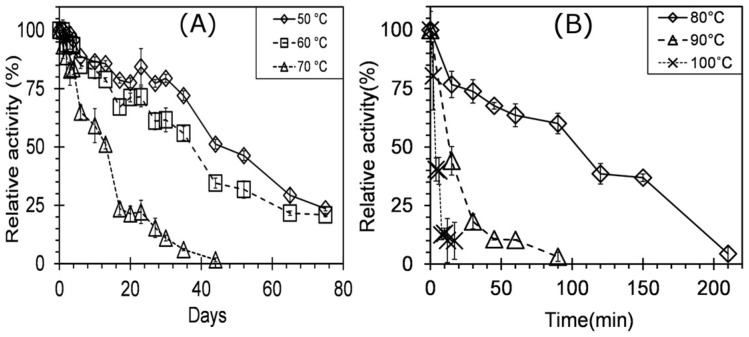
Thermal stability profiling of the xylanase produced by *Geobacillus* sp. strain DUSELR13 at different temperatures (**A**) 50–70 °C and (**B**) 80–100 °C. The enzyme activity is expressed as the percentage of the initial activity that is taken as 100%. The values are mean of triplicate experiments and the error bars represent ± SD of the means with *n* = 3.

The thermostability of the DUSELR13 xylanase was determined by incubating it at different temperatures. The xylanase retained >50% of the activity at temperatures 50, 60, and 70 °C for 48, 38, and 13 days respectively (Figure 8A,B). The xylanase was highly thermostable and retained >23.5% of activity at 50 °C after 75 days. At 60 and 70 °C respectively, it retained 21 and 6% activity after 75 and 44 days. However, when the xylanase was incubated at 80, 90, and 100 °C respectively, resulted in the loss of 50% of its activity in 110, 18, and 5 min. The thermostable xylanase from *Geobacillus* sp. strain WSUCF1 retained 70% of its activity after 19 days at 50 °C [5]. Interestingly, other thermostable xylanases did not retain their activity for long time periods. The xylanase from *Geobacillus thermodenitrificans* strain A333 retained 50% of the activity for 60 min at 70 °C [4], *Geobacillus* sp. strain TC-W7 retained 80% of the activity for 90 min at 70 °C [41], and *Geobacillus thermodenitrificans* strain T12 retained only 18% of the initial activity for 60 min at 70 °C [16]. The xylanases XynA1 and XynA2 from *Geobacillus thermodinitrificans* strain NG80-2 reported by Huang and coworkers showed >50% activity at 65 °C for 24 h [43]. However, as the temperature was increased to 75 °C XynA1 and XynA2 respectively showed only 15% activity after 4 h and 15 min [43]. The other xylanase with such high thermostability had been reported from *Geobacillus* sp. strain WSUCF1, which retained 50% of enzymatic activity for 19 and 12 days respectively at 60 and 70 °C [5]. Thus, the xylanase from *Geobacillus* sp. strain DUSELR13 is highly thermostable among the known xylanases and can be of promising use in high temperature bioprocesses that run for longer durations.

**Figure 9:**
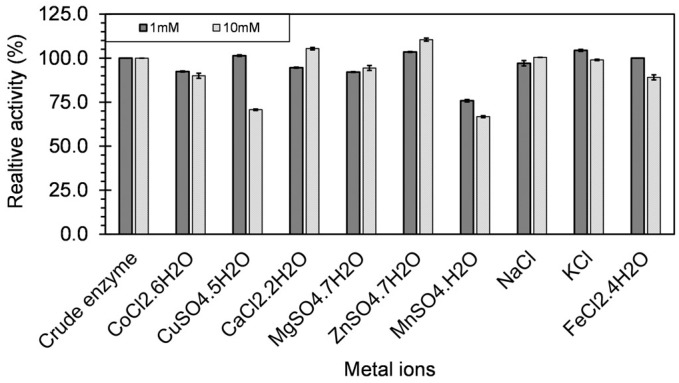
The effect of metal ion on the xylanase activity of *Geobacillus* sp. strain DUSELR13. The activity of the crude xylanase is taken as 100% (30.6 U/mL) and effect of the metal ions on the xylanase activity is expressed as the percentage of the initial activity. The values are mean of triplicate experiments and the error bars represent ± SD of the means with *n* = 3.

The enzyme activity is often affected by the presence of metal ions in the complex substrate (e.g., food waste, agricultural and forestry residues), or the medium. The metal ions either serve as the catalytic center of the enzymes, acts as the bridge to bring the enzyme and substrate in close proximity or maintain other physiological functions [45]. Hence, the effect of different metal ions on the xylanase activity was determined at 1mM and 10mM concentrations (Figure 9). The xylanase activity on the crude xylanase grown with xylan as the substrate without any external metal ion addition was taken as 100%, and all the relative activities were measured against it. At 1 mM concentration the addition of Cu^+2^, Zn^+2^, K^+^, and Fe^+2^ showed an increase in the endoxylanase activity, but Na^+^, Mg^+2^, and Ca^+2^ resulted in the decrease of xylanase activity. However, at 10mM Ca^+2^, Zn^+2^, Na^+^, and Mg^+2^ had a positive effect on the xylanase activity, but Cu^+2^, K^+^, and Fe^+2^ reduced the activity of the xylanase. Co^+2^ and Mn^+2^ decreased the xylanase activity at both the concentrations, but the observed decrease was more with Mn^+2^ as the concentration increased from 1 mM to 10 mM. Maximum increase in the xylanase activity, with the increase in the metal ion concentration from 1mM to 10mM, was observed with addition of CaCl_2_, where the relative activity increased from 94.6% to 105.7%. On the contrary, maximum decrease was observed with the addition of CuSO_4_, where it decreased from 101.4% to 70.7%. 

Several literature studies have shown varying effects of metal ions on the xylanase activity at different concentrations. For xylanase produced by *Geobacillus thermodenitrificans* strain A333 with increase in the concentration from 1mM to 10mM, the addition of Na^+^, Cu^+2^, Mg^+2^, and Fe^+2^ respectively decreased the xylanase activity from 122% to106%, 79% to 76%, 72% to 70% and 80% to 3%; whereas addition of K^+^ increased the xylanase activity from 142% to 145%, and Ca^+2^ and Zn^+2^ had no effect [4]. Two xylanases XynA1, and XynA2 obtained from *Geobacillus thermodenitrificans* strain NG80-2 showed different behavior towards the Zn^+2^ metal ion. In the presence of 5 mM Zn^+2^ the activity of XynA1 increased, whereas with XynA2 for it decreased [43]. The activity of xylanase produced by *Cladosporium oxysporum* increased after the addition of Mg^+2^ and Ca^+2^ (1 mM), but was inhibited after adding Cu^+2^ (1 mM) [44]. Similarly, increased enzymatic activity was observed by the addition of Ca^+2^, Cu^+2^, Co^+2^, and Mn^+2^ (1 mM), but the activity was inhibited by the addition of Cd^+2^, Hg^+2^, Ba^+2^, Mg^+2^, Fe^+2^ (1 mM) in case of xylanase produced by *Caldiprobacter algeriensis* sp. no. strain TH7C1 [46]. Thus the different concentrations of metal ions have variable effect on the xylanase activity, even for enzymes obtained from similar organism, and hence the metal ion concentration needs to be tightly regulated.

### 3.4. Hydrolysis of the Beechwood Xylan

**Figure 10:**
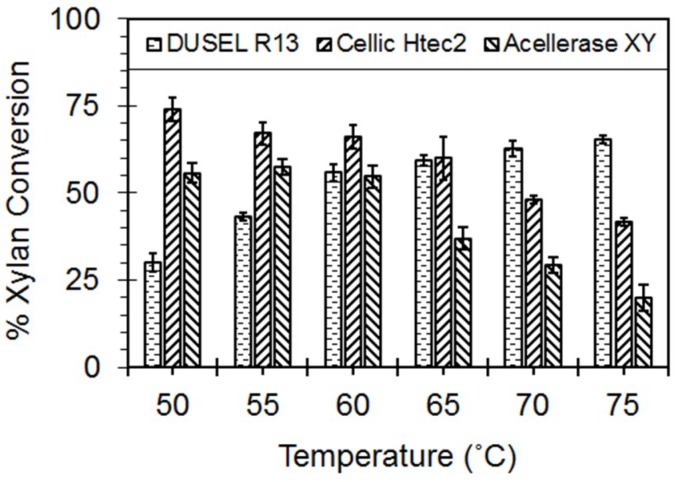
Hydrolysis of the beechwood xylan by DUSELR13, and comparative analysis of the hydrolytic activity with Cellic HTec2 and Accellerase XY. The percentage xylan conversion is calculated on the basis of the sugar released from 1 g of xylan, with complete hydrolysis of xylan considered as 100%. The values are mean of triplicate experiments and the error bars represent ± SD of the means with *n* = 3.

The thermostable enzymes have been considered with potential to improve the current high temperature fermentative industrial processes. The hydrolytic potential of the xylanase from DUSELR13 was compared to its commercial counterpart Cellic Htec2 [47] and Accellerase XY at their operationally stable conditions. Accellerase XY has an operational stability in the range of 50–75 °C, and pH 4.5 and 7.0 [48], and Cellic HTec 2 has also showed activity at a temperature range of 50–75 °C, and pH 4.5 and 6.75. Figure 10 shows the hydrolysis of the beechwood xylan by the DUSELR13 crude xylanase, and its commercial counterparts. The percentage conversions were calculated on the basis of reducing sugars released from 1 g beechwood xylan. The maximum amount of sugar that can be obtained with xylan hydrolysis under ideal conditions where complete hydrolysis occurs was taken as 100%. The xylanase from DUSELR13 converted more xylan into sugars at high temperature whereas the commercial counterparts showed higher percentage of xylan hydrolysis at relatively lower temperatures. At 75 °C, DUSEL R13 xylanase hydrolyzed 65.4% of the beechwood xylan, whereas Cellic HTec2, and Accellerase XY respectively showed only 41.8%, and 20% hydrolysis. However, at lower temperature the hydrolysis by DUSELR13 was lower in comparison to that obtained with Cellic HTec2, and Accellerase XY. At 50 °C, the xylanase from DUSELR13 showed only 30% xylan hydrolysis in comparison to 74%, and 55.8% respectively by Cellic HTec2 and Accellerase XY. The hydrolysis rate for DUSELR13 increased significantly from 55 to 60 °C, but the change was not that significant from 60 to 75 °C. This was also observed during the T_opt_ for the xylanase activity in Figure 5B, where the increase in the xylanase activity showed similar trend. On the other hand, the xylanase activity for Cellic HTec2 reduced significantly from 55 to 75 °C. With increase in temperature to 75 °C, the xylan hydrolysis by DUSELR13 increased 1.52 times to 65.4%, however, for Cellic HTec2, and Accellerase XY respectively it reduced 1.77 times to 41.8%, and 2.79 times to 20%. 

Bhalla and coworkers (2015) also reported that the highly thermostable xylanase from *Geobacillus* sp. strain WSUCF1 performed better than the commercial counterparts at higher temperatures. At 70 °C, WSUCF1 xylanase yielded higher conversions of 68.9% as compared to Cellic HTec2 (49.4%) and Accellerase XY (28.92%). However, the commercial counterparts also outperformed WSUCF1 xylanase at lower temperatures (T = 50 °C), suggesting that the xylanase from WSUCF1 also performed better at high temperature processes [5]. Similarly, the thermostable xylanase obtained from *Thermotoga themarum* hydrolyzed 87% of the beechwood xylan at 85 °C after 3 h of incubation [10]. The present hydrolysis results show that the xylanase from DUSELR13 was better than the commercial counterpart at high temperatures. The high temperature activity, and thermostability of the crude enzyme cocktail from DUSELR13 will be suitable for use in several high temperature industrial processes that involve direct lignocellulosic biomass utilization.

### 3.5. Enzyme Production with Lignocellulosic Biomass

**Figure 11:**
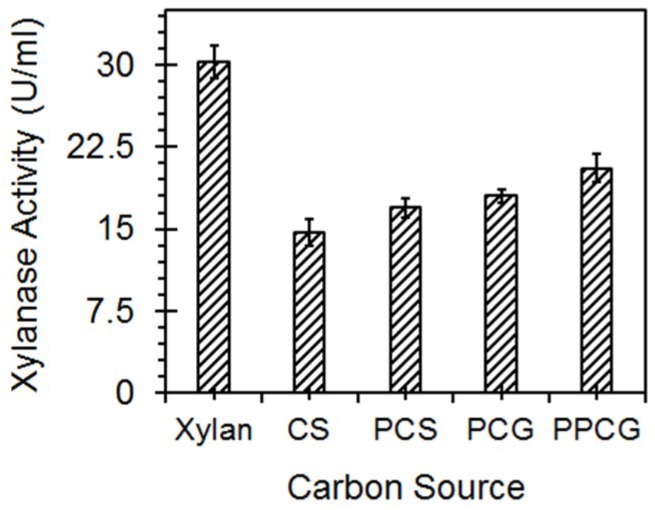
Enzyme production with untreated and mechanically treated corn stover and prairie cordgrass with comparison to xylan under optimized conditions. The values are mean of triplicate experiments and the error bars represent ± SD of the means with *n* = 3.

The use of lignocellulosic biomass for enzyme production is economically advantageous due to its cheap and ubiquitous availability. DUSELR13, was able to use both untreated and mechanically pretreated lignocellulosic biomass for xylanase production. The order of the endoxylanase activity with different carbon sources as the substrate was: xylan (100%: 30.2 U/mL) > pretreated prairie cordgrass—PPCG (67.9%) > untreated prairie cordgrass—PCG (59.5%) > pretreated corn stover—PCS (56%) > untreated corn stover—CS (48.6%) (Figure 11). DUSELR13 showed more enzyme activity when it grew on the mechanically pretreated lignocellulosic biomass, and the xylan, as these sources provide easily accessible carbon for growth in comparison to the untreated lignocellulosic biomass. However, its capability to use the untreated substrates substantiates its industrial potential for enzyme production, using the cheaper carbon sources without pretreatment. The xylanase production using lignocellulosic biomass had been done previously also with *Streptomyces termitum* using bagasse, straw sugarcane, and cocoa pod husk [9] for xylanase production; *Geobacillus* sp. stain WSUCF1 utilizing corn stover and prairie cordgrass for the same [5]; and *Caldicellulosiruptor owensensis* using corn cob [49]. Corn stover and prairie cordgrass used in this study are inexpensive lignocellulosic biomasses that do not compete with food resources for human. CS had been extensively used in several bioprocesses: biobutanol, bioflocculant, bioethanol; and lipid [50]. Prairie cordgrass also had been previously reported in the production of enzymes, and biofuels [5,25]. The availability of a cheaper resource viz. lignocellulosic biomass that can be used in the industrial bioprocesses for providing fermentable sugars is very important. The organisms that can utilize such substrates hold significance, and DUSELR13 is one such strain that can be put into use for this purpose. 

## 4. Ethanol Production with Lignocellulosic Biomass

**Figure 12:**
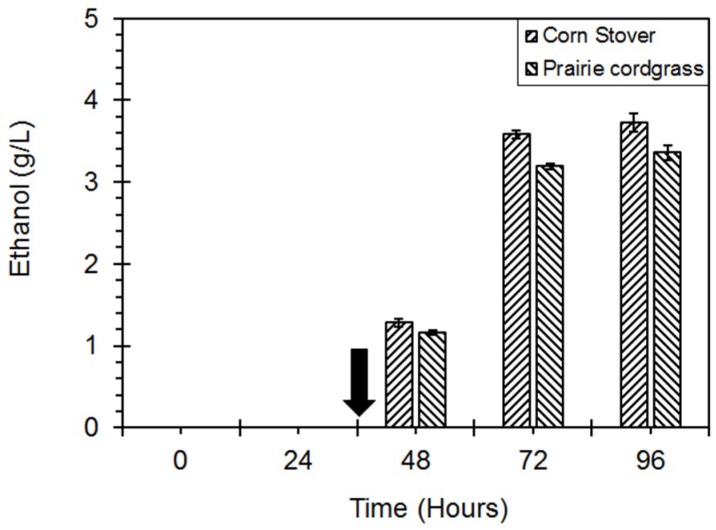
Ethanol production with co-culture of *Geobacillus* sp. strain DUSELR13, and *Geobacillus thermoglucosidasius*. The black arrow represents the point of addition of *Geobacillus thermoglucosidasius.* The values are mean of triplicate experiments and the error bars represent ± SD of the means with *n* = 3.

Five percent (*w*/*v*) of thermo-mechanically treated insoluble corn stover and prairie cordgrass [25] were used for ethanol production in 500-mL Erlenmeyer flasks. The flasks were inoculated with *Geobacillus* sp. strain DUSELR13 at time 0 h, and with *Geobacillus thermoglucosidasius* at time 36 h. An ethanol yield of 3.53 g/L (0.21 g/g PCG utilized) and 3.72 g/L (0.2 g/g CS utilized), respectively were obtained with prairie cordgrass and corn stover (Figure 12). The material balance showed that 90.5 and 91.6% of the mass was recovered during the process, converting 62.4 and 57.4% energy respectively in prairie cordgrass and corn stover to ethanol (Table 5). The ethanol productivity obtained was 0.059 (g/L/h) and 0.62 (g/L/h) with Corn Stover and prairie cordgrass, respectively. 

*Geobacillus* sp. strain DUSELR13 had been previously known for producing thermostable cellulase [24], and this study presents the production of a thermostable endoxylanase by this organism. The presence of cellulase and hemicellulases can aid in the hydrolysis of the lignocellulosic biomass, producing the sugars required for fermentation. After the addition of *Geobacillus thermoglucosidasius*, the ethanol production was observed. Along with ethanol, acetate, lactate, and propionate were also produced as the main fermentation products showing that the fermentation was mixed acid type. The ethanol production observed with corn stover was higher than that observed with prairie cordgrass. This can be attributed to the compositional difference among the two substrates. Corn stover has lower lignin content (16%) as compared to prairie cordgrass (21%) [51]. *Geobacillus thermoglucosidasius* known for ethanol production lacks lignocellulosic biomass hydrolyzing enzymes [13], and hence cannot hydrolyze the lignocellulosic biomass, whereas on the other hand *Geobacillus* sp. strain DUSELR13 is unable to produce ethanol. Hence, the ethanol production was observed only when the two cultures were grown together. None of the monocultures showed ethanol production with lignocellulosic biomass. Park et al. (2012) also observed that the co-culture of cellulase producing *Acremonium cellulolyticus* and ethanologen *Saccharomyces cerevisiae* produced 8.7 g/L with 5% (*w*/*v*) steam solka-floc as susbtrate [52]. Similar results were also observed by Miyazaki et al. (2008) where co-cultures of aerobic cellulolytic *Geobacillus* sp. strain kpuB3 aided in ethanol production by anaerobic hemicellulolytic *Thermoanerobacterium* sp. strain kpu04 using bean curd refuse [53]. The co-culture of *Geobacillus* sp. strain kpuB3 and *Thermoanerobacterium* sp. strain kpu04 produced a total of 1.26 g/L ethanol whereas the monoculture of *Thermoanaerobacterium* sp. strain kpu04 produced only 0.37 g/L ethanol.

## 5. Conclusions

A wild-type thermophilic microorganism, like *Geobacillus* sp. strain DUSELR13 which can utilize the lignocellulosic biomass and produce thermostable enzymes, was studied in the present work. The DUSELR13 produced 6 U/mL of a highly thermostable xylanase, which after optimization was increased to 31 U/mL. The xylanase showed better hydrolytic potential than the commercial counterparts with xylan at higher temperatures. The enzyme was active over a wide range of pH and temperatures and had the potential to utilize and produce the xylanase using lignocellulosic biomass (e.g., corn stover and prairie cordgrass). The hydrolytic potential of the xylanase with lignocellulosic biomass helped to hone its characteristics for bioethanol production in co-culture studies with *Geobacillus thermoglucosidasius.* The DUSELR13 strain has already been researched for cellulase, and now this study further substantiates its use for the lignocellulosic biomass hydrolysis at higher temperatures for future biotechnological improvements.

## Figures and Tables

**Table 1:** Experimental range, level, and coded representation of independent variables for the CCD design.

Variables	Code	Range and Levels				
		−1	−0.5	0	+0.5	+1
Temperature (°C)	A	55	57.5	60	62.5	65
pH	B	6	6.25	6.5	6.75	7
Xylan (g/L)	C	5	7.5	10	12.5	15
Tryptone (g/L)	D	1	3.25	5.5	7.75	10

**Table 2:** Central composite design along with experimental and predicted values of the dependent variable.

Run	A	B	C	D	Xylanase Activity (IU/mL)	
					Experimental	Predicted
1	1.000	1.000	−1.000	1.000	13.60	15.06
2	0.500	0.000	0.000	0.000	26.40	24.92
3	−1.000	−1.000	1.000	1.000	17.60	17.51
4	1.000	1.000	−1.000	−1.000	12.00	11.75
5	0.000	0.000	0.000	0.000	24.70	24.93
6	0.500	0.000	0.000	0.000	16.30	15.80
7	−1.000	−1.000	1.000	−1.000	16.10	15.06
8	−1.000	1.000	1.000	−1.000	23.40	23.24
9	−1.000	1.000	1.000	−1.000	23.10	24.23
10	−0.500	0.000	0.000	0.000	26.20	24.23
11	1.000	1.000	1.000	1.000	18.70	19.83
12	1.000	−1.000	−1.000	1.000	27.50	26.72
13	0.000	0.000	0.000	0.000	20.00	22.51
14	1.000	−1.000	−1.000	−1.000	18.30	17.51
15	1.000	−1.000	1.000	−1.000	26.20	24.92
16	0.000	0.000	0.000	−0.500	15.70	15.80
17	1.000	−1.000	1.000	1.000	15.60	15.16
18	0.000	0.000	0.500	0.000	22.40	24.23
19	−1.000	1.000	−1.000	−1.000	12.30	12.21
20	0.000	0.500	0.000	0.000	19.30	19.48
21	0.000	0.500	0.000	0.000	18.40	19.08
22	0.000	0.000	0.000	−0.500	16.30	16.51
23	0.000	0.000	0.000	0.500	22.60	21.35
24	1.000	1.000	1.000	1.000	23.20	25.89
25	0.000	−0.500	0.000	0.000	18.40	19.83
26	−1.000	1.000	−1.000	1.000	18.60	19.48
27	1.000	−1.000	1.000	1.000	23.80	23.24
28	1.000	1.000	1.000	−1.000	13.30	12.52
29	0.000	0.000	0.000	0.000	15.30	15.06
30	0.000	0.000	0.000	0.000	27.00	24.92
31	0.000	0.000	−0.500	0.000	17.30	16.57
32	−1.000	−1.000	1.000	−1.000	21.20	19.83
33	−1.000	1.000	1.000	1.000	23.10	23.97
34	0.500	0.000	0.000	0.000	23.70	25.89
35	1.000	1.000	1.000	−1.000	22.50	23.40
36	−1.000	1.000	1.000	1.000	21.30	23.24
37	1.000	1.000	−1.000	1.000	16.80	17.85
38	−1.000	1.000	−1.000	−1.000	18.30	17.85
39	−1.000	−1.000	−1.000	−1.000	15.30	15.16
40	1.000	−1.000	−1.000	1.000	14.80	15.16
41	−0.500	0.000	0.000	0.000	19.60	19.48
42	0.000	0.000	0.000	0.500	14.50	13.17
43	−1.000	−1.000	−1.000	−1.000	13.10	12.52
44	0.000	0.000	0.000	−0.500	16.50	16.57
45	1.000	−1.000	−1.000	1.000	15.80	16.51
46	1.000	1.000	−1.000	−1.000	23.40	22.51
47	1.000	−1.000	−1.000	−1.000	26.10	24.92
48	1.000	−1.000	−1.000	−1.000	17.10	16.57
49	0.000	0.000	0.000	0.000	12.10	12.52
50	−1.000	−1.000	−1.000	1.000	12.30	12.21
51	1.000	1.000	1.000	1.000	26.20	24.93
52	0.000	0.000	0.500	0.000	22.00	23.40
53	−1.000	−1.000	1.000	−1.000	24.50	25.89
54	1.000	1.000	−1.000	−1.000	11.20	11.75
55	0.000	0.000	0.000	0.500	28.40	26.72
56	−1.000	−1.000	−1.000	−1.000	13.00	12.44
57	−1.000	−1.000	−1.000	1.000	13.30	13.17
58	0.000	0.500	0.000	0.000	24.50	23.97
59	0.000	0.000	−0.500	0.000	19.00	19.08
60	−0.500	0.000	0.000	0.000	16.00	16.51
61	−1.000	−1.000	−1.000	1.000	17.40	17.85
62	−1.000	1.000	−1.000	−1.000	16.80	17.51
63	−1.000	1.000	1.000	−1.000	20.90	21.35
64	0.000	0.000	0.000	0.000	26.80	24.92
65	0.000	0.000	−0.500	0.000	11.20	11.75
66	−1.000	1.000	1.000	1.000	14.80	15.80
67	−1.000	−1.000	1.000	1.000	12.40	13.17
68	1.000	−1.000	1.000	−1.000	21.30	22.51
69	−1.000	−1.000	1.000	1.000	25.40	24.92
70	−1.000	1.000	−1.000	1.000	21.80	21.35
71	0.000	−0.500	0.000	0.000	24.20	23.97
72	0.000	−0.500	0.000	0.000	13.10	12.44
73	1.000	−1.000	1.000	−1.000	24.50	24.93
74	0.000	0.000	0.500	0.000	12.80	12.21
75	−1.000	1.000	−1.000	1.000	28.30	26.72
76	1.000	1.000	1.000	−1.000	23.60	23.40
77	1.000	−1.000	1.000	1.000	20.40	19.08
78	1.000	1.000	−1.000	1.000	12.20	12.44

**Table 3:** ANOVA for the xylanase activity as a function of independent variables as obtained in the simulation. The main factor effects, and two factor interaction effects having an effect on the xylanase activity are shown.

Source	Sum of Squares	df	Mean Square	F Value	*p*-Vale	Prob > F
**Model**	0.0734	14	0.0052	146.69	<0.0001	significant
A-Temperature	0.0177	1	0.0177	496.16	<0.0001	
B-pH	0.0024	1	0.0024	68.47	<0.0001	
C-Xylan	0.0010	1	0.0010	27.96	<0.0001	
D-Tryptone	0.0023	1	0.0023	63.44	<0.0001	
AB	0.0003	1	0.0003	7.64	0.0075	
AC	0.0000	1	0.0000	0.5490	0.4615	
AD	0.0017	1	0.0017	48.19	<0.0001	
BC	0.0001	1	0.0001	3.89	0.0529	
BD	0.0003	1	0.0003	8.63	0.0046	
CD	0.0001	1	0.0001	3.37	0.0711	
A²	0.0003	1	0.0003	8.57	0.0047	
B²	0.0003	1	0.0003	7.45	0.0082	
C²	0.0003	1	0.0003	7.51	0.0080	
D²	0.0002	1	0.0002	4.55	0.0368	
**Residual**	0.0023	63	0.0000			
Lack of Fit	0.0005	10	0.0001	1.61	0.1297	Not significant
Pure Error	0.0017	53	0.0000			
**Cor Total**	0.0756	77				

R^2^: 0.9702, adj R^2^: 0.9636, predicted R^2^: 0.9538, C.V. 2.56% adeq precision 38.13; df = degree of freedom; cor = correlation, Highly significant, *p* ≤ 0.0001; Significant, *p* ≤ 0.05; non-significant, *p* ≥ 0.05.

**Table 4:** The characteristics of various thermostable xylanases.

Organism	Type	Enzyme Activity (U/mL)	M.Wt. ^#^ (kDa)	T_opt_ (°C)	pH_opt_	Thermostability	Reference
*Geobacillus* sp. strain DUSELR13	Wild type	31.0	~45	75	7.0	t_1/2_ = 13 days at 70 °C	This Study
*Geobacillus* sp. strain WSUCF1	Wild type	23.8	37	70	6.5	t_1/2_ = 12 days at 70 °C	[5]
*Geobacillus thermolevorans*	Wild type	26.52	~45	80	8.5	t_1/2_ = 50–55 min at 80 °C	[6]
*Geobacillus thermodinitrificans* strain A333	Wild type	0.02	44	70	7.5	t_1/2_ = 60 min at 70 °C	[4]
*Geobacillus thermodinitrificans* strain NG80-2 XynA1	Recombinant	40.4	N.A.*	70	7.6	t_1/2_ = 28 h at 65 °C	[43]
*Geobacillus thermodinitrificans* strain NG80-2 XynA2	Recombinant	36.8	N.A.	70	6.5	t_1/2_ = 26 h at 65 °C	[43]
*Geobacillus* sp. strain TF16	Recombinant	7.92	38.9	55	8.5	t_1/2_ = 10 min at 70 °C	[17]
*Bacillus amyloliquefaciens*	Wild type	48.5	~50	50	9.0	t_1/2_ = 45 min at 70 °C	[23]
*Bacillus pumilus* SV-85S	Wild type	2995	N.A.	50	6.0	t_1/2_ = 25 min at 70 °C	[7]
*Cladosporium oxysporum* GQ-3	Wild type	55.92	N.A.	50	8.0	t_1/2_ = 30 min at 70 °C	[44]
*Malbranchea pulchela*	Wild type	3.0	49	80	5.5	t_1/2_ = 260 min at 70 °C	[8]

^#^ M.Wt. Molecular weight. * N.A. Not available.

**Table 5:** Mass balance for ethanol production using PCG and CS.

Substrate/Metabolite	Mass Balance			
	PCG	%	CS	%
	Mass		Mass *	
Substrate Utilized	13.8	100	16.7 ^#^	100
Biomass	-	-	-	-
Acetate	1.46	10.57971	1.68	10.05988
Lactate	4.12	29.85507	5.66	33.89222
Propionate	0.37	2.681159	0.64	3.832335
Ethanol	3.35	24.27536	3.72	22.27545
CO_2_	3.2	23.18841	3.6	21.55689
Total	12.5		11.7	
Recovery		90.57971		91.61677

* g/L. ^#^ Amount of substrate utilized.
